# The effectiveness of teaching complementary and alternative medicine based on the components of theory of planned behavior on nutrition students: multicenter research study

**DOI:** 10.1186/s12909-023-04239-8

**Published:** 2023-04-17

**Authors:** Mohammad Reza Mahmoodi, Sara Shafian, Manizhe Shaban Alinaghizade

**Affiliations:** 1 Physiology Research Center, Institute of Neuropharmacology, Kerman University of Medical Sciences, Haft Bagh-E-Alavi Highway, Kerman, 7616913555 Iran; 2 Department of Nutrition, Faculty of Public Health, Kerman University of Medical Sciences, Kerman, Iran; 3 Department of Medical Education, Education Development Center, Kerman University of Medical Sciences, Kerman, Iran

**Keywords:** Complementary and alternative medicine, Effectiveness of teaching, Theory of planned behavior

## Abstract

**Objective:**

Safe and beneficial use of complementary and alternative medicine (CAM) modalities results from integrating CAM education into curricula and increasing CAM knowledge. We sought how much teaching CAM procedures in a virtual environment can influence the components of the theory of planned behavior (TPB), including knowledge, attitude, behavioral intention, and behavior of nutrition students.

**Methods:**

This cross-sectional descriptive-analytic study was conducted on 162 undergraduate nutrition students were selected through convenience sampling based on inclusion criteria in 2021–2022. Data were collected through a validated researcher-made CAM questionnaire that was designed based on TPB questionnaire that consisted of four constructs (knowledge, attitude, behavioral intention, and behavior). The content validity index and content validity ratio of the questionnaire were evaluated.

**Results:**

Our results revealed that students who significantly scored the highest scores in attitude, behavioral intention, and behavior constructs were the students who claimed that “teaching two credits of CAM for nutrition students is not enough” (*p*. value =  < 0.001, < 0.001, and 0.005, respectively). In addition, these students used treatment methods of CAM for themselves, suggested treatment methods of CAM to others, and followed the recommendations of the CAM specialists (for three pairwise comparisons: *p*. value =  < 0.001, < 0.001, and < 0.001, respectively). The attitude could predict 70% of behavioral intention. However, attitude and behavioral intention could predict 90% of behavior.

**Conclusion:**

Attitude was the most critical determinant influencing behavioral intention and behavior. CAM teaching using evidence-based CAM-ITM principles with a succinct, concerted, and collaborative curriculum, integration of CAM into continuing education, and integration of educational CAM programs continuously for several years into the academic curriculum in the actual setting influence the success of the educational CAM program.

## Introduction

Complementary and alternative medicine (CAM) modalities are entirely favorite in Iran, among which Iranian Traditional Medicine (ITM) and Acupuncture are the most used. As an eastern country, Iran needs to communicate with East Asian countries to institute academic relations on ITM [1]. The results of a survey revealed that CAM is the most widely used in three East Asian countries such as Japan (76%), South Korea (75%), and Malaysia (56%) [2].

The CAM integration in conventional healthcare services enhances the capacity of holistic services and results in filling therapeutic gaps in available healthcare procedures, treating the patient, and increasing healthcare choices [3]. Regarding the extensive use of CAM, practitioners and health system managers should pay more attention to CAM modalities. Thereby, the supervision and training of traditional practitioners and nutritionists are critical [4]. Regulation of academic standards and the development of consistent curricula for CAM will help students proficiently provide safe and efficient CAM therapies [5]. Safe and beneficial use of CAM modalities results from integrating CAM education practices into curricula and increasing knowledge of CAM modalities [6]. Integration of CAM training into the medical curriculum remarkably influences the attitude of medical students toward CAM. The capability of communicating with patients about safe and confident CAM use is the primary motivation of medical students in CAM learning [7]. The knowledge of the students regarding ITM and CAM was low. The student’s attitude toward the integration of ITM and CAM into their curriculum was positive. The majority of the students had not passed any courses on ITM. Therefore, university policymakers should provide diverse training courses on ITM and CAM to increase students' knowledge [8].

Results of some studies revealed that pharmacy, medical, and nursing students had positive attitudes and perceptions toward CAM therapies, despite poor knowledge regarding CAM modalities [9–12]. All students agreed and supported the integration of CAM education into the medical education curriculum due to its importance concerning the doctor-patient encounter and advising patients regarding safe and beneficial CAM use [11–14]. On the other hand, people of some communities were very interested in CAM practices and had good knowledge, but their attitude towards CAM was low [15]. Familiarization and acclimatization of health professionals with ITM and CAM as an inseparable part of Iran's heritage can help them select the best remedy options for their patients. Hence, authorities have been suggested including ITM and CAM into the medical education curricula of medical and some paramedical students as a two-credit course for implementation [16].

Nutrition students commonly must have desirable knowledge and attitudes toward CAM as adjuvant therapy to treat patients under diet therapies to have proficiency and competency in counseling patients regarding the interaction between diet and CAM therapies. Hence, due to the lack of research studies regarding the effectiveness of CAM education on nutrition students, we sought how much teaching CAM procedures in a virtual environment can influence the components of the theory of planned behavior (TPB) including their knowledge, attitude, behavioral intention, and behavior of nutrition students.

## Materials and methods

### Participants eligibility, sampling and study design

This cross-sectional descriptive-analytic study was conducted on 162 undergraduate nutrition students were selected through convenience sampling based on inclusion criteria in 2021–2022 from 13 universities. Sixty four nutrition students aged 22–25 years old from Kerman University of Medical Sciences and 98 nutrition students from 12 other universities of medical sciences enrolled in this study. The inclusion criteria were 1) nutrition students who have passed two-credit CAM in a virtual environment supported through the Virtual University that cover teaching medical and some paramedical students from summer 2021 in Iran; 2) willingness to participate in the study; and 3) ability to understand the educational content and participate in the study; the exclusion criterion was 1) unwillingness to continue cooperation due to any reason. Some of nutrition students refused to cooperate due to a lack of motivation. Indifference in presenting the opinion to change the imposed educational curriculum is also one of the other reasons for not cooperating in the study. Data was collected through a validated researcher-made CAM questionnaire. Before the start of the study, the purpose of the project and the concealment of the information was explained to them. After getting permission and coordinating with the nutrition students, the questionnaires were sent through Email to every student in a routine manner. The students completed the questionnaires and referred them to the researchers. To maximize the internal validity of the study data and prevent information bias, we decided that all nutrition students who have taken this course in a certain period in a virtual setting will be assessed. The method of choosing participants was convenience sampling which is a specific type of non-probability sampling method that relies on data collection from University students who are conveniently available to participate in the study. Therefore, the goal was to include all nutrition students from medical sciences universities in the country who were willing to cooperate with our study.

Careful administration of the study was done by the original executor from the Kerman University of Medical Sciences. Monitoring of the good implementation of the study was done by selected supervisors from the National Agency for Strategic Research in Medical Education. The Research Ethics Committee of the National Agency for Strategic Research in Medical Education approved the protocol (Approval ID: IR.NASRME.REC.1400.455). This project was funded by the National Agency for Strategic Research in Medical Education; Tehran, Iran (Grant No. 4000432).

### Designing of CAM questionnaire and questionnaire validation

The structured researcher-made CAM questionnaire was designed based on the TPB questionnaire that consisted of educational variables, knowledge queries, and the TPB constructs. The modified CAM-TPB constructs of the structured questionnaire were designed based on a modified 5-point Likert scale which consisted of 34 items in 4 subscales under the following titles: knowledge (10 items), attitude (11 items), behavioral intention (7 items), and behavior (6 items). Knowledge means the theoretical or practical understanding acquired through education and teaching. However, attitude is defined as an individual's favorable or unfavorable evaluation of a determined behavior. On the other hand, we designed four two-level queries for attitude construct to better interpret the data of four constructs. Therefore, these four two-level queries for attitude were selected as part of the statistical analysis of the data. A 5-point Likert scale with 1–5 coding was used for the knowledge construct: very much = 5, much = 4, Intermediate = 3, low = 2, and none = 1. On the Likert scale, strongly disagree = 1 and strongly agree = 5 was used for the attitude construct. On the Likert scale of behavioral intention, very low probability = 1 and very high probability = 5; and on the Likert scale of behavior, never = 1 and always = 5.

Five experts familiar with medical education and nutrition determined the face and content validities. The content validity index (CVI) and content validity ratio (CVR) of the CAM-TPB questionnaire were evaluated. The CVI was calculated at 0.98 for the overall scale. The reliability was calculated by measuring the internal consistency and test–retest method (12-day interval). Only 51 students out of 64 students who freely entered the study from Kerman University of Medical Sciences agreed to participate in the validation of the questionnaire. To measure internal consistency, Cronbach's alpha coefficient was calculated for 51 samples. The Intra Class Correlation (ICC) was calculated. The range of Cronbach's alpha coefficient for the four constructs was 0.847–0.943.

### Statistical analysis

Data were analyzed using IBM SPSS Statistics software, version 22.0. Significance was assumed at *P* < 0.05. The normal distribution of variables was examined by the Kolmogorov–Smirnov test. The Chi-square test was used to determine the relationship between the gender variable and three different levels (favorable, relatively favorable, and unfavorable) of four constructs. In Table 2, to avoidance of confusion and a more precise interpretation of collected data, the lower tertile of the whole score of each construct is considered unfavorable. The middle tertile of the whole score of each construct is considered relatively favorable. The upper tertile of the whole score of each structure is considered desirable. Therefore, overall score grouping was based on unfavorable, relatively favorable, and favorable. The Chi-square test was used to determine the relationship between gender variable and two-level queries of attitude construct. Independent t-test analyzed the mean differences of four constructs (knowledge, attitude, behavioral intention, and behavior) scores between the pairwise comparisons of the attitude construct. The Pearson correlation coefficient was calculated to examine the relationship between the mean scores of four constructs. Linear regression analysis was used to predict the value of a variable (the dependent variable) based on the values of the other variables. At first, the behavioral intention was the dependent variable, and knowledge and attitude were the independent variables. In the second step, behavior was the dependent variable, and knowledge, attitude, and behavioral intention were the independent variables.

## Results

The mean (± SD) age of male students was 22.64 ± 1.53 years and for female students was 22.32 ± 1.30 (34% males and 66% females).

The Chi-Square test analyzed the differences between frequencies of responses to the pairwise comparisons of the attitude construct based on two genders (Table 1). The percentage of students that responded to the phrase “two credits of the CAM course were adequate for teaching nutrition students” did not differ by gender, X^2^ (1, *N* = 162) = 2.158, *p* = 0.171. The percentage of “nutrition students that used CAM modalities for themselves” did not differ by gender, X^2^ (1, *N* = 162) = 0.004, *p* = 1.000. The percentage of “nutrition students that recommended CAM modalities to others” did not differ by gender, X^2^ (1, *N* = 162) = 0.344, *p* = 0.613. The percentage of “nutrition students that would follow the recommendation of a CAM specialist” did not differ by gender, X^2^ (1, *N* = 162) = 0.848, *p* = 0.376.

**Table 1:** Frequencies^a^ of the pairwise comparisons^b^ of the attitude^c^ based on two genders.

Genders	**Adequacy of CAM**^**1**^			
	**YES (** ***n*** ** = 61)**	**NO (** ***n*** ** = 101)**	**Pearson Chi-Square**	***Sig. (2sided)***
Male	25(45.5)	30(54.5)	2.158	0.171
Female	36(33.6)	71(66.4)		
	**Use of therapeutic methods of CAM ** ^**2**^			
	**YES (** ***n*** ** = 86)**	**NO (** ***n*** ** = 76)**	**Pearson Chi-Square**	***Sig. (2sided)***
Male	29(52.7)	26(47.3)	0.004	1
Female	57(53.3)	50(46.7)		
	**Suggest of therapeutic methods of CAM ** ^**3**^			
	**YES (** ***n*** ** = 64)**	**NO (** ***n*** ** = 98)**	**Pearson Chi-Square**	***Sig. (2sided)***
Male	20(36.4)	35(63.6)	0.344	0.613
Female	44(41.1)	63(58.9)		
	**Recommend of therapeutic methods of CAM** ^**4**^			
	**YES (** ***n*** ** = 110)**	**NO (** ***n*** ** = 51)**	**Pearson Chi-Square**	***Sig. (2sided)***
Male	35(63.6)	20(36.4)	0.848	0.376
Female	75(70.8)	31(29.2)		

^a^Chi-Square test analyzed the differences between the pairwise comparisons of the attitude construct based on two genders

^b^Pairwise comparisons generally are methods for comparing entities in pairs to judge which of each entity is preferred or has a greater amount of some quantitative property, or whether or not the two entities are identical

^c^ Two-level queries of attitude such as 1) Are two credits of the CAM course adequate for teaching students? 2) Have you ever used CAM modalities for yourself? 3) Have you ever recommended CAM modalities to others? 4) If a CAM specialist recommends one of the modalities, would you use it?

The Chi-Square test also analyzed the differences between whole scores grouping (unfavorable, relatively favorable, and favorable) for four constructs (knowledge, attitude, behavioral intention, and behavior) based on two genders (Table 2). The percentage of whole scores grouping did not differ by gender for knowledge construct, X^2^ (2, *N* = 162) = 4.814, *p* = 0.090; attitude construct, X^2^ (2, *N* = 162) = 0.995, *p* = 0.608; behavioral intention, X^2^ (2, *N* = 162) = 0.349, *p* = 0.840; behavior, X^2^ (2, *N* = 162) = 0.656, *p* = 0.720 (Table 2).

**Table 2:** Frequencies^a^ of total scores grouping^b^ for four constructs (knowledge, attitude, behavioral intention, and behavior) between two genders.

Genders	**Knowledge scores grouping**				
	**Unfavorable**	**Relatively favorable**	**Favorable**	**Pearson Chi-Square**	***Sig. (2sided)***
Male	28(50.9)	25(45.5)	2(3.6)	4.814	0.09
Female	36(33.6)	63(58.9)	8(7.5)		
	**Attitude scores grouping**				
	**Unfavorable**	**Relatively favorable**	**Favorable**	**Pearson Chi-Square**	***Sig. (2sided)***
Male	4(7.3)	26(47.3)	25(45.5)	0.995	0.608
Female	4(3.7)	54(50.5)	49(45.8)		
	**Behavioral intention scores grouping**				
	**Unfavorable**	**Relatively favorable**	**Favorable**	**Pearson Chi-Square**	***Sig. (2sided)***
Male	9(16.4)	29(52.7)	17(30.9)	0.349	0.84
Female	14(13.1)	60(56.1)	33(30.8)		
	**Behavior scores grouping**				
	**Unfavorable**	**Relatively favorable**	**Favorable**	**Pearson Chi-Square**	***Sig. (2sided)***
Male	7(12.7)	37(67.3)	11(20.0)	0.656	0.72
Female	10(9.3)	78(72.9)	19(17.8)		

^a^Chi-Square test analyzed the differences between total scores grouping for four constructs (knowledge, attitude, behavioral intention, and behavior) based on two genders

^b^Whole score grouping was based on unfavorable, relatively favorable, and favorable. The lower third of the whole score of each construct is considered unfavorable. The middle third of the whole score of each construct is considered relatively favorable. The upper third of the whole score of each structure is considered desirable.

There was a significant difference between the total score of knowledge in the two genders (*p*. value = 0.031). However, there were no significant differences between both gender groups in total scores of attitude, behavioral intention, and behavior (Table 3).

**Table 3:** Mean ± SD^a^ of total scores of four constructs (knowledge, attitude, behavioral intention, and behavior) between two genders.

Constructs	**Male (** ***n*** ** = 55)**	**Female (** ***n*** ** = 107)**	***Sig***	***t-value***
Total scores of Knowledge construct	18.09 ± 7.43	20.99 ± 8.33	**0.031**	-2.174
Total scores of Attitude construct	34.40 ± 9.61	35.02 ± 7.91	0.662	-0.438
Total scores of Behavioral Intention construct	19.84 ± 6.92	19.75 ± 6.79	0.938	0.078
Total scores of Behavior construct	16.71 ± 5.11	16.45 ± 5.21	0.762	0.303

^a^Independent t-test analyzed the differences (Mean ± SD) of total scores of four constructs (knowledge, attitude, behavioral intention, and behavior) between two genders

Table 4 indicated that students who claimed “two credits of the CAM course were not adequate for teaching” earned significantly more scores in attitude, behavioral intention, and behavior (*p*. value =  < 0.001, < 0.001, and 0.005, respectively). The students who claimed that they “used CAM modalities for themselves” earned significantly more scores in attitude, behavioral intention, and behavior (*p*. value =  < 0.001, < 0.001, and < 0.001, respectively). The students who claimed that they “recommended CAM modalities to others” earned significantly more scores in knowledge, attitude, behavioral intention, and behavior (*p*. value = 0.003, < 0.001, < 0.001, and < 0.001, respectively). The students who claimed that they “would follow the recommendation of a CAM specialist” earned significantly more scores in knowledge, attitude, behavioral intention, and behavior (*p*. value = 0.001, < 0.001, < 0.001, and < 0.001, respectively) (Table 4).

**Table 4:** Mean ± SD^a^ four constructs (knowledge, attitude, behavioral intention, and behavior) scores between the pairwise comparisons^b^ of the attitude^c^.

	**Adequacy of CAM** ^**1**^			
Constructs	**YES (** ***n*** ** = 61)**	**NO (** ***n*** ** = 101)**	***Sig***	***t-value***
Knowledge	20.28 ± 8.83	19.84 ± 7.72	0.741	-0.331
Attitude	30.57 ± 8.84	37.37 ± 7.20	** < 0.001**	5.071
Behavioral Intention	17.33 ± 6.85	21.26 ± 6.39	** < 0.001**	3.692
Behavior	15.10 ± 5.53	17.41 ± 4.75	**0.005**	2.815
	**Use of therapeutic methods of CAM** ^**2**^			
	**YES (** ***n*** ** = 86)**	**NO (** ***n*** ** = 76)**	***Sig***	***t-value***
Knowledge	21.16 ± 8.15	18.70 ± 7.96	0.054	-1.942
Attitude	37.81 ± 6.42	31.41 ± 9.29	** < 0.001**	-5.043
Behavioral Intention	21.92 ± 6.13	17.36 ± 6.78	** < 0.001**	-4.499
Behavior	18.52 ± 4.31	14.29 ± 5.16	** < 0.001**	-5.694
	**Suggest of therapeutic methods of CAM** ^**3**^			
	**YES (** ***n*** ** = 64)**	**NO (** ***n*** ** = 98)**	***Sig***	***t-value***
Knowledge	22.31 ± 8.63	18.50 ± 7.46	**0.003**	-2.988
Attitude	39.73 ± 5.85	31.59 ± 8.44	** < 0.001**	-7.254
Behavioral Intention	24.19 ± 5.34	16.90 ± 6.12	** < 0.001**	-7.791
Behavior	19.53 ± 4.10	14.58 ± 4.87	** < 0.001**	-6.737
	**Recommend of therapeutic methods of CAM** ^**4**^			
	**YES (** ***n*** ** = 110)**	**NO (** ***n*** ** = 51)**	***Sig***	***t-value***
Knowledge	21.44 ± 8.55	16.92 ± 6.28	**0.001**	-3.371
Attitude	38.73 ± 5.44	26.37 ± 7.85	** < 0.001**	-10.169
Behavioral Intention	22.59 ± 5.57	13.78 ± 5.26	** < 0.001**	-9.498
Behavior	18.73 ± 4.12	11.92 ± 3.98	** < 0.001**	-9.845

^a^Independent t-test analyzed the differences (Mean ± SD) of four constructs (knowledge, attitude, behavioral intention, and behavior) scores between the pairwise comparisons of the attitude construct

^b^Pairwise comparisons generally are methods for comparing entities in pairs to judge which of each entity is preferred or has a greater amount of some quantitative property, or whether or not the two entities are identical

^c^ Two-level queries of attitude such as 1) Are two credits of the CAM course adequate for teaching students? 2) Have you ever used CAM modalities for yourself? 3) Have you ever recommended CAM modalities to others? 4) If a CAM specialist recommends one of the modalities, would you use it?

The Pearson correlation coefficient between attitude and behavioral intention as well as between attitude and behavior were strong (*r* = 0.764, *p* < 0.001; *r* = 0.762, *p* < 0.001). The Pearson correlation coefficient between behavioral intention and behavior was strong (*r* = 0.862, *p* < 0.001) (Table 5).

**Table 5:** Pearson correlation coefficients for the relationship between mean scores of four constructs.

Theory of Planned Behavior Constructs	Total scores of Knowledge construct	Total scores of Attitude construct	Total scores of Behavioral Intention construct	Total scores of Behavior construct
**Total scores of Knowledge construct**	1	0.367	0.423	0.415
		< 0.001	< 0.001	< 0.001
**Total scores of Attitude construct**		1	0.764	0.762
			< 0.001	< 0.001
**Total scores of Behavioral Intention construct**			1	0.862
				< 0.001
**Total scores of Behavior construct**				1

Table 6 revealed that the association between the behavioral intention and predictors of knowledge and attitude in the model is statistically significant. However, attitude contributes the most to the variability in behavioral intention. The higher the R^2^ value, the better the model fits our data.

**Table 6:** Linear regression analysis for predicting behavioral intention from knowledge and attitude based on theory of planned behavior.

Model^a^	Unstandardized coefficients	Standardized coefficients				
						Adjusted R square
	B	Beta	Sig	R	R square	
(Constant)	-2.637		0.075	0.78	0.608	0.603
Knowledge	0.138	0.164	0.002			
Attitude	0.565	0.704	0			

^a^Dependent variables: Behavioral intention; Predictors: Knowledge and attitude

Table 7 revealed that the association between the behavior and predictors of attitude and behavioral intention in the model is statistically significant. The p-value for knowledge was higher than 0.05, which indicated that there was not enough evidence to conclude that knowledge related to the behavior. However, behavioral intention contributes the most to the variability in the behavior. The higher the R^2^ value, the better the model fits our data.

**Table 7:** Linear regression analysis for predicting behavior from knowledge, attitude, and behavioral intention based on theory of planned behavior.

Model^a^	Unstandardized coefficients	Standardized coefficients				
						Adjusted R square
	B	Beta	Sig	R	R square	
(Constant)	0.962		0.266	0.878	0.771	0.766
Knowledge	0.3	0.048	0.258			
Attitude	0.147	0.243	0			
Behavioral intention	0.497	0.656	0			

^a^Dependent variables: Behavior; Predictors: Knowledge, attitude, and behavioral intention

## Discussion

Nutrition students customarily must have favorable knowledge and attitudes toward CAM as an adjuvant therapy to treat patients under therapeutic diets to have expertise and competency in counseling patients regarding the interaction between diet and CAM therapies. Hence, we sought how much teaching CAM procedures in a virtual environment can influence the components of the TPB including knowledge, attitude, behavioral intention, and behavior of nutrition students. Our results revealed that there was no difference in gender between the pairwise comparisons of the attitude construct (Yes/No). Furthermore, the percentage of whole scores grouping did not differ by gender for knowledge, attitude, behavioral intention, and behavior constructs. The students who significantly scored the highest scores in attitude, behavioral intention, and behavior constructs were the students who claimed that teaching two credits of CAM for nutrition students is not enough. In addition, these students used treatment methods of CAM for themselves, suggested treatment methods of CAM to others, and followed the recommendations of the CAM specialists.

Results of some studies revealed that pharmacy, medical, and nursing students had positive attitudes and perceptions toward CAM therapies, despite poor knowledge concerning CAM modalities [8–12]. Therefore, university policymakers should provide diverse training courses on ITM and CAM to increase student’s knowledge [8]. The data also suggested a significant association between familiarity with CAM and gender [9]. In this regard, we revealed that there was a significant difference between the total score of knowledge in the two genders.

We used adjusted R square to compare models that had different numbers of predictors to choose the correct model. It appears the second model is the best. Attitude could predict 70% of behavioral intention. However, attitude and behavioral intention could predict 90% of behavior. Therefore, attitude is the most critical determinant influencing behavioral intention and behavior.

**Fig. 1:**
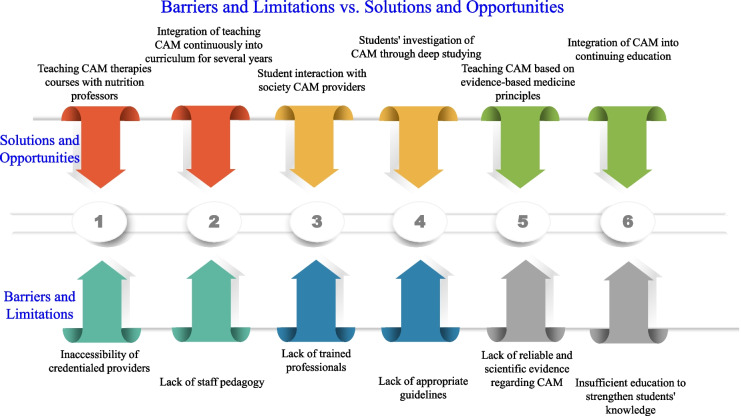
Major barriers and limitations to obstacle CAM education-included curricula; and solutions and opportunities which can eliminate these barriers

In this context, a question is raised as that why in most of the studies [6–8, 11–15], all students agreed and supported the integration of CAM education into the medical education curriculum; nonetheless, despite poor knowledge regarding CAM modalities, the majority of students had positive attitudes and perceptions about CAM usage. Therefore, the reasons for the gap between students’ knowledge and attitude must be determined. The findings indicate that integration of CAM into the nursing curriculum and continuing education/training in CAM is necessary to prepare future nurses [11]. It proved that the lack of trained professionals, inaccessibility of credentialed providers, lack of staff pedagogy, lack of appropriate guidelines, and lack of reliable and scientific evidence regarding CAM were the most apperceived obstacles to CAM execution [11, 17]. One of the weaknesses of CAM education-included curricula was how to strengthen students' knowledge [18]. The solution to this weakness that is educational CAM programs should be integrated continuously for several years into the educational curriculum to increase students' knowledge and performance [19]. The timing of the CAM curriculum near graduation, students' investigation of several CAM modalities through deep studying, and student interaction with society CAM providers are features of the curriculum that make the curriculum prosperous and unforgettable [20]. Figure 1 illustrates the crucial barriers and limitations to obstacle CAM education-included curricula; and solutions and opportunities which can eliminate these barriers. CAM education-included curriculum influences the knowledge and attitude of students concerning CAM and advises patients concerning CAM use, and the practice of CAM therapies in the future [20]. Most students were uncertain about the effectiveness of herbs in promoting good health. The students claimed that obstacles to their CAM knowledge were insufficient education, a lack of scientific evidence, and a lack of trained professionals and education. Most students requested CAM educational courses regarding CAM therapies with nutrition professors being ranked highest as the providers of this education [21]. Medical educators must be efficiently capable of teaching CAM using evidence-based medicine principles with a concise, interactive curriculum. This procedure has a good impetus for medical educators and a useful influence on medical students' willingness to gain new knowledge regarding CAM [22]. Training of holistic nurses and practitioners depend on CAM integration study into medical curricula and the practice of the CAM therapies to promote their knowledge. They demonstrated the effectiveness of CAM education for the remarkable development in the preparation of holistic practitioners and nurses [23]. The majority of the students agreed that CAM therapy could integrate with modern medicine to achieve better consequences. The knowledge of CAM is important since many patients still prefer this module of treatment [24]. Therefore, the CAM integration in conventional healthcare services, familiarization and acclimatization of health professionals with ITM and CAM, the supervision and training of traditional practitioners and nutritionists, increase proficiency and competency in counseling patients regarding the interaction between diet and CAM therapies, regulation of academic standards, and the development of consistent curricula for CAM can enhance the capacity of holistic healthcare services.

### Strengths and limitations

The most important strength of this study is the implementation of this research in nutrition students and their behavior rationalization through the most effective educational model. One of the limitations of this study is the non-participation of all nutrition students in the present study. Two critical reasons were unwillingness to participate in the study for personal reasons and failure to teach this course to nutrition students in some universities of medical sciences in the country.

## Conclusions

We concluded that the prerequisite for obtaining expertise and competency in counseling patients regarding the integration between diet and CAM therapies is to have desirable knowledge and attitudes toward CAM. The attitude could predict 70% of behavioral intention. The attitude and behavioral intention together could predict 90% of behavior. Therefore, the attitude was the most important determinant influencing behavioral intention and behavior. Teaching CAM using evidence-based CAM-ITM principles with a succinct, concerted, and collaborative curriculum and integration of educational CAM programs continuously for several years into the academic curriculum influence the success of the educational CAM program. The removing obstacles on the way to knowledge increase such as the lack of trained professionals, inaccessibility of credentialed providers, and lack of reliable and scientific evidence regarding CAM.

## Data Availability

The data that support the findings of this study are available from the corresponding author, but restrictions apply to the availability of these data, which were used under license for the current study, and so are not publicly available.
